# A contribution to the study of plant development evolution based on gene co-expression networks

**DOI:** 10.3389/fpls.2013.00291

**Published:** 2013-08-05

**Authors:** Francisco J. Romero-Campero, Eva Lucas-Reina, Fatima E. Said, José M. Romero, Federico Valverde

**Affiliations:** ^1^Department of Computer Science and Artificial Intelligence, Universidad de SevillaSevilla, Spain; ^2^Molecular Plant Development and Metabolism, Instituto de Bioquímica Vegetal y Fotosíntesis, Consejo Superior de Investigaciones Científicas y Universidad de SevillaSevilla, Spain

**Keywords:** photoperiod, evolution, gene co-expression networks, *Chlamydomonas*, *Physcomitrella*, *Arabidopsis*

## Abstract

Phototrophic eukaryotes are among the most successful organisms on Earth due to their unparalleled efficiency at capturing light energy and fixing carbon dioxide to produce organic molecules. A conserved and efficient network of light-dependent regulatory modules could be at the bases of this success. This regulatory system conferred early advantages to phototrophic eukaryotes that allowed for specialization, complex developmental processes and modern plant characteristics. We have studied light-dependent gene regulatory modules from algae to plants employing integrative-omics approaches based on gene co-expression networks. Our study reveals some remarkably conserved ways in which eukaryotic phototrophs deal with day length and light signaling. Here we describe how a family of *Arabidopsis* transcription factors involved in photoperiod response has evolved from a single algal gene according to the innovation, amplification and divergence theory of gene evolution by duplication. These modifications of the gene co-expression networks from the ancient unicellular green algae *Chlamydomonas reinhardtii* to the modern brassica *Arabidopsis thaliana* may hint on the evolution and specialization of plants and other organisms.

## Introduction

Day length, or photoperiod, regulates strategic developmental processes in plants. It constitutes a key external signal to feed the circadian rhythms with the needed information to maintain the set of the day (Imaizumi, 2010) and is also critical to discern seasons (Jackson, 2009). Thus, photoperiodic signals regulate crucial developmental responses such as dormancy; germination; senescence or the transition from vegetative to reproductive stages (Valverde, 2011). The floral transition is one of the most conserved evolutionary processes in angiosperms (Romero and Valverde, 2009; Serrano et al., 2009) due to its central role in producing new plant generations and in the transmission of acquired characteristics. Many of the key genes involved in the floral transition are conserved in an inter-species dependent manner as well as many of the external cues that trigger the reproductive response (Amasino, 2010). According to this, the response to the three most important external agents that control reproduction, namely temperature, nutrients and day length, are regulated by a set of inter-species gene regulatory networks with conserved functions (Ausín et al., 2005).

The photoperiodic flowering pathway involves a series of genes that are influenced by light and circadian signals to orchestrate a response that ensures the best moment of the year to flower. Thus, long-day (LD) plants flower as the day lengthens and short-day (SD) plants flower when days start to shorten. In the model species *Arabidopsis*, a facultative LD plant, a detailed knowledge of the gene pathways involved in the photoperiod floral transition has been accumulated and solid mechanisms have been proposed to explain its flowering behavior (Fornara et al., 2010). In this pathway, the role of the gene *CONSTANS* (*CO*) is crucial because its fine control at several regulatory levels (Valverde et al., 2004) assure that the plant triggers the flowering response exactly at the precise moment of the year. It modulates the expression of the florigen *FLOWERING LOCUS T* (*FT*) gene in the leaf vasculature, whose protein, reaching the meristem, triggers the reproductive developmental program, eventually producing flowers (Corbesier et al., 2007). While many genes have been discovered in this regulatory module, dealing with protein stability (Jang et al., 2008; Lázaro et al., 2012), modulating the light response of the proteins (Yu et al., 2008) or their function (Kim et al., 2007), the integration of the pathway within other flowering routes or the output genes that trigger the flowering transition are less known. Due to the characteristic attached-to-the-soil behavior of plants, a complex network of regulatory processes is in the base of their physiological responses. These complex systems can be better analysed employing holistic and integrative approaches (Usadel et al., 2009; Tohge and Fernie, 2012).

The last years have seen a bloom of massive data acquisition techniques to approach plant biology in a holistic and integrative way, particularly in transcriptional information (Metzker, 2010). Microarrays first and Next-Generation Sequencing (NGS) later have provided enormous amount of gene expression data for a multitude of plant species in different physiological/genotypic conditions and developmental stages (Schliesky et al., 2012). These data can also be enriched by further experimental approaches and include this information into the gene co-expression networks generated. Therefore, when trying to analyze the developmental response of a plant to an external condition we can combine data from gene co-expression analyses generated using microarray and/or NGS approaches with physiological data such as the time to flower, the weight of the plant or the chlorophyll content. This helps to associate a particular plant behavior to a particular gene expression pattern. Thus, the levels of complexity poised by the enormous amounts of data generated at different regulatory levels, such as transcriptomics, proteomics and metabolomics can be approached with an integrative and holistic perspective employing network tools to reduce noise and find novel patterns of organization. When an evolutionary approach covering the history of gene networks within the phylogeny of organisms is employed, an interesting relation between function and diverse processes, such as developmental processes, starts to be unveiled. This could help to explain many of the intriguing intertwines between evolution and development observed in different organisms (Müller, 2007).

Sequence similarity constitutes the classical approach to assign potential functions to genes and to study their evolutionary history (Lajoie et al., 2010). This methodology focuses on the comparison between individual genes and do not take into account that genes perform their function in coordination with many other genes. In this respect, sequence similarity has been shown to be incomplete when predicting phenotypic differences between species. For example, the genes involved in the human and chimpanzee brains share very high sequence similarities that do not correspond with the marked phenotypic differences between them (Oldham et al., 2006). Holistic and integrative approaches such as gene co-expression networks that take into account the orchestration among genes are emerging as powerful tools to predict gene function and to infer their evolutionary history. This methodology assumes that if a group of genes are co-expressed they should have similar functions and a common evolutionary history. In these networks, genes are represented as nodes and an edge is established between two nodes if the expression profiles of the corresponding genes exhibit a correlation value high enough to provide evidence of co-expression. The topological analysis of these networks such as the distribution of the number of neighbors of each gene, the number of co-expressed genes for a given gene, can provide information about their function and evolutionary history (Aoki et al., 2007; Usadel et al., 2009).

In this paper, we have studied the evolution of the co-expression sub-networks or modules around the photoperiod central family of *CO-Like* genes, *CONSTANS (CO)* homologs, in three model species whose genome is available: the green unicellular alga *Chlamydomonas reinhardtii* (*CrCO* gene), the moss *Physcomitrella patens* (*PpCOL* genes) and the higher plant *Arabidopsis thaliana* (*AtCOL* genes). These species are landmarks of the evolutionary lineage of plants. Microarrays or RNA-seq database experiments have been used to construct gene co-expression networks. Phylogenetic analyses have been combined together with functional enrichment analyses in order to better understand the evolution of the *CO-Like* (*COLs*) family between the species and their functional specialization. This combination of gene expression data analysis, gene ontology (GO) term enrichment and phylogenetic studies constitutes a novel methodology to study gene function and evolution. The approach is not restricted to our case study and can be applied to the study of sub-networks or modules of other transcription factors. Through the analysis of the evolution of gene networks we can study gene duplication and diversification and how this has affected the networks. Additionally, our methodology could be a useful tool to identify homologous genes related to the same specific process and therefore predict true gene orthology. Finally, our analysis can also be used to explain why and to what extent different signaling pathways are linked in an organism and, therefore, constitute a valuable tool to understand plant plasticity.

## Materials and methods

### Plant, algal material, and growth conditions

*Arabidopsis thaliana* 35S:*CO*-GR (Simon et al., 1996) transgenic lines were grown in MS plates. Seeds were previously incubated 4 days at 4°C in the dark before sowing under 16 h light/8 h dark cycle (long-day, LD) with temperature ranging from 22°C (day) to 18°C (night) at 75 μE/m^2^ light intensity. In the experiment employing dexamethasone (DEX) 1 μg/mL and cycloheximide (CHX) 1mM chemicals were added 10 days after sowing at ZT0 and leaf samples harvested 4 h after the drug treatment, considering Zeitgeber Time 0 (ZT0) the moment at which the lights are switched on.

*Chlamydomonas reinhardtii* cell-wall deficient mutant CW15 (Davies and Plaskitt, 1971) and pNIA:*CrCO* (Serrano et al., 2009) transgenic line were grown in stirred conical cylindrical flasks containing Sueoka NO^−^_3_ (Sueoka et al., 1967) under LD conditions in control rooms at 22°C and 50 μE/m^2^. Algal cells were harvested at 4 days at ZT4, 4 h after the lights went on. Plants and algae were grown in a model SG-1400 phytotron (Radiber SA, Spain).

### RNA isolation and Q-PCR

RNA was isolated from *Arabidopsis* seedlings (0.1 g leaf tissue) and *Chlamydomonas* (20 ml of an exponential phase culture) employing, in both cases, the TRIZOL (Invitrogen) protocol as described by the manufacturer. In short, the sample was mixed with 1ml of TRIZOL and 0.2 ml of chloroform, the mixture was then centrifuged at 16,000 g for 10 min at 4°C. The supernatant was treated with 1 volume of 2-propanol, incubated 15 min at room temperature and centrifuged at 16,000 g for 10 min at 4°C. 0.75 ml 3 M LiCl was added to the pellet and incubated for *t* >10 min at room temperature and centrifuged at 16,000 g for 10 min at 4°C. The pellet was washed with 80% (v/v) ethanol and centrifuged at 16,000 g for 10 min at 4°C. The final RNA sample was suspended in 30 μl of DEPC treated water and quantified employing a ND-1000 Spectrophotometer (Nanodrop).

1 μg of TRIZOL isolated RNA was used to synthesize cDNA employing the Quantitec® Reverse kit (Qiagen) following the instruction recommended by the manufacturer. cDNA was diluted to a final concentration of 10 ng/μL and stored at −20°C until Q-PCR was performed. Primers to amplify the 3′ translated region of *AtCO, AtSSS, AtFAD, AtGS, AtZEP, AtWRKY33, AtUBQ10, CrCO, CrSSS, CrFAD, CrGS, CrZEP*, *CrATG8*, and *CrTUB* (Table 1) were designed employing an Oligo analyzer program (Integrated DNA technologies, http://eu.idtdna.com/analyzer/Applications/OligoAnalyzer/). Q-PCR was performed in a Multicolor Real-Time PCR Detection System iQTM5 from Bio-Rad in a 10 μL reaction: primer concentration 0.2 μM, 10 ng cDNA and 5 μL SensiFAST TM SYBR & Fluorescein Kit (Bioline). Each sample was measured by triplicate. The Q-PCR program consisted in (1) 1 cycle (95°C, 2 min); (2) 40 cycle (95°C, 5 s; 60°C for *Arabidopsis* primers and 65°C to *Chlamydomonas* primers, 10 s and 72°C, 6 s) (iii) 1 cycle (72°C, 6 s). Fluorescence was measured at the end of each extension step and the melting curve was performed between 55 and 95°C. The initial concentration of candidate and reference gene was calculated by means of LingRegPCR software version 11.0 (Ruijter et al., 2009). Normalized data was calculated dividing the average of four replicates of each sample of the candidate and reference genes.

**Table 1:** **Primers employed for Q-PCR experiments**.

**Arabidopsis genes**	**Sequence**	**Amplified fragment size (bp)**
CO	5′ -CCAATGGACAGAGAAGCCAGG-3′ 5′ -GCATCGTGTTGAACCCTTGC- 3′	175
AtSSS	5′ -CTGGGGATCATCAGCTACACAATACG-3′ 5′ -CACGTGCGATTAGGAACAGCTC-3′	81
AtFAD	5′- CGTCGTTAAGTTCCTTCAAGCC -3′ 5′- CATAGCTTCAATCGAACCGACAG -3′	157
AtGS	5′- CCAGCTTCGAACATGGATCC -3′ 5′-CCTAAGACATTGCTTGATAGAGAACAC-3′	167
AtZEP	5′- CTCCGAAATCGACGAGGAAG -3′ 5′- TGCAAGGAATAGCTGAAAGCAG -3′	166
AtUBQ10	5′- GAAGTTCAATGTTTCGTTTCATGT -3′ 5′- GGATTATACAAGGCCCCAAAA -3′	119
AtERF	5′- CCAATGTTCAGCAGAATGCC -3′ 5′- GGACGATGAGAAAGAATTAGGAG -3′	85
AtWRKY33	5′-TACCGGGCCTTTTGGTTA-3′ 5′- CCACCACCAACAAAGTTTTG-3′	81
**Chlamydomonas genes**	**Sequence**	**bp**
CrCO	5′- CTTCCCGCAAGGCGTATGC -3′ 5′- GCCTCAATCTCCTCCTTCTTGGC -3′	73
CrSSS	5′- ACGTGTACCGCTCCATCAGC -3′ 5′- GCAGCACTCTTGCACTATGCAG -3′	107
CrFAD	5′- GACGAGAAGGTCAACTACAAGCC -3′ 5′- GCTTGCTCAGCTCCGATTAGC -3′	150
CrGS	5′- GCTACGGCTACCTGGAGGA -3′ 5′- CATCGCTGCCCTTATTAGCTGG -3′	127
CrZEP	5′- AAGAGGCAGGTTGGCTTAGTGC -3′ 5′- GGTGTCTGTCAACGTGTGTAGC -3′	127
CrTUB	5′- GTTGCATCGTTAGCGTGGACG -3′ 5′- GCAGCAGCCAATGTTCAGACT -3′	170
CrERF	5′- AGCCAGGCTCGCTGCAACTTCC -3′ 5′- GGA AGT TGC AGC GAG CCT GGC T -3′	108
CrATG8	5′- TCCCGATATCGACAAGAAG -3′ 5′- TGCGGATGACGTACACAAAT -3′	75

### Phylogenetic analysis

Evolutionary relationships among the *CO-Like* genes of *Chlamydomonas*, *Physcomitrella* and *Arabidopsis* were analyzed using predicted amino-acid sequences from Phytozome v9.0 and aligned with the program MUSCLE (Edgar, 2004). This alignment was then used to generate a phylogenetic tree applying the Neighbor-Joining algorithm (Saitou and Nei, 1987) with the JTT+G 1.53 as substitution model (Jones et al., 1992). The bootstrap consensus tree inferred from 1000 replicates is taken to represent the evolutionary history of the analyzed proteins. The tree is drawn to scale, with branch lengths in the same units as those of the evolutionary distances used to infer the phylogenetic tree. Phylogenetic analyzes were conducted with MEGA5 (Tamura et al., 2011). Accession numbers of sequences used in the alignment are shown in Table 2.

**Table 2:** **COL protein family in *Clamydomonas, Physcomitrella*, and *Arabidopsis* employed in the phylogenetic studies of this work**.

**Protein**	**Gene number**	**Protein**	**Gene number**
CrCO	g6302	CO	At5g15840
PpCOL1	Pp1s371_27V6	AtCOL1	At5g15850
PpCOL2	Pp1s97_109V6	AtCOL2	At3g02380
PpCOL3	Pp1s364_5V6	AtCOL3	At2g24790
PpCOL4	Pp1s36_238V6	AtCOL4	At5g24930
PpCOL5	Pp1s26_5V6	AtCOL5	At5g57660
PpCOL6	Pp1s236_21V6	AtCOL6	At1g68520
PpCOL7	Pp1s195_82V6	AtCOL7	At1g73870
PpCOL8	Pp1s143_52V6	AtCOL8	At1g49130
PpCOL9	Pp1s108_97V6	AtCOL9	At3g07650
PpCOL10	Pp1s3_491V6	AtCOL10	At5g48250
		AtCOL11	At4g15250
		AtCOL12	At3g21880
		AtCOL13	At2g47890
		AtCOL14	At2g33500
		AtCOL15	At1g28050
		AtCOL16	At1g25440

The left column includes the short protein code number as found in the literature (PpCOL4-PpCOL10 named in this work) and the right column the gene code number from the corresponding genome databases (Phytozome and TAIR).

### Identification of conserved motifs

The sequences of COL proteins from the different plants and algae were analyzed using the MEME program (http://meme.nbcr.net/meme/) as described by Bailey et al. (2009). Only the three main motifs (2 B-boxes and CCT domain) were represented. The motifs were characterized using the Conserved Domain Search Service (http://www.ncbi.nlm.nih.gov/Structure/cdd/wrpsb.cgi) (Marchler-Bauer and Bryant, 2004).

### Data compilation and processing

This study is based on an ensemble of more than one TeraByte transcriptomic data from *Chlamydomonas*, *Physcomitrella*, and *Arabidopsis* obtained under related physiological and genotype conditions (Table 3). These data comprise RNA-seq experiments from the public database, the Sequence Read Archive (SRA, http://www.ncbi.nlm.nih.gov/Traces/sra/) (Wheeler et al., 2005) and microarray data from the Array Express Archive (ArrayExpress, http://www.ebi.ac.uk/arrayexpress/) (Rustici et al., 2013). For *Chlamydomonas* 50 RNA-seq data sets representing eight different genotypes under diverse physiological conditions (González-Ballester et al., 2010; Miller et al., 2010; Castruita et al., 2011; Kropat et al., 2011; Boyle et al., 2012; Fischer et al., 2012; Urzica et al., 2012) were analyzed. For *Physcomitrella* 13 RNA-seq data sets representing two different genotypes (Zemach et al., 2010; Xiao et al., 2011; Chen et al., 2012) were analyzed. Finally, in order to use comparable conditions *Arabidopsis* microarray data from experiments under similar conditions to those from *Chlamydomonas* and *Physcomitrella* (Gutiérrez et al., 2007; Long et al., 2010; Patterson et al., 2010; Iyer-Pascuzzi et al., 2011; Cheng et al., 2013) were analyzed. The experimental conditions analyzed in this study are diverse enough to capture the true co-expression among genes. These conditions include nutrient deficiency (nitrogen, iron, copper, and sulfur deprivation), oxidative stress, light stimuli, DNA damage and different developmental stages (Table 3).

**Table 3:** **Description of the experiments and datasets employed in this work**.

**Experiment**	**Accession number**	**Data base**
2137 WT cells were cultivated photoheterotrophically under Fe-replete (20 mM), Fe-deficient (1 mM) and Fe-limited (0.25 mM) conditions	SRP010563	SRA
2137 WT cells were exposed to hydrogen peroxide for 0, 0.5, and 1 h	SRP010084	SRA
4A+ WT cells and *sor1* mutant cells were grown in 12 h light/dark cycle for several days. Samples were taken at ZT6	SRP009273	SRA
2137 WT cells and *crr1* mutant cells were cultivated in TAP or minimal medium under Cu-sufficient and Cu-defficient conditions	SRP005483	SRA
CW15 cells were cultivated in N-repleted, N-deprived conditions	SRP003630	SRA
D66 WT cells and *ars11* mutant cells were cultivated under continuous light in S-repleted and S-deprived conditions	SRP002284	SRA
Dark grown WT and double *pubs*/*hy2* mutant protonema were irradiated with red light for 1 h	SRP011279	SRA
WT protonemal, caulonemal, and chloronemal tissues were collected at 3, 14, 24, 30 days of development	SRP009201	SRA
Samples were collected in control tissues and tissues treated with the DNA-DSB inducing agent bleomycin	SRP004443	SRA
Col-0 WT and *pye* mutant seedlings were grown in Fe-repleted and Fe-deprived media	GSE21582	GEO
Col-0 plants were grown hydroponically, transferred to a nitrogen free medium for 26 h and finally supplied with 1 mM nitrate or 1 mM ammonium	GSE29589	GEO
Col-0 plants were hydroponically in nutrient solutions with various concentrations of nitrate and sucrose	E-MEXP-828	Array express
Col-0 seedlings were cultivated in the dark to reduce endogenous H_2_O_2_ then were treated with 5mM H_2_O_2_	GSE40574	GEO
Five days old Col-0 seedlings were transferred to sulfur deficient media. Samples were collected at 0, 3,12, 24, 18, and 72 h	GSE30098	GEO
Samples from Col-0, *CO* overexpressor 35S:*CO* and *co-2* mutant were collected in long-day conditions (16 h light/8 h dark) at ZT4	E-MTAB-1078	Array express

The columns represent the species name, accession numbers and data bases acronyms for the different experimental datasets. A short descriptive paragraph of the experimental design (as stated by the authors in the databases) is also provided.

An estimation of gene expression in the transcriptomes generated in the different conditions under study was obtained following the methodology described in Trapnell et al. (2012). This approach uses a reference genome for each species. The *Chlamydomonas* reference genome corresponds to the release v5.3 which is assembled into 17 chromosomes and 37 additional unmapped scaffolds. The *Chlamydomonas* genomic information is based on the Augustus update u11.6. For *Physcomitrella* we used the reference genome release v1.6 which is assembled into 2106 scaffolds that have not been mapped yet to the 27 chromosomes that constitute its genome. The *Physcomitrella* genomic information is based on the Cosmoss update v1.6. Finally, the *Arabidopsis* genomic information is based on the TAIR v10 resource. This annotation and genome information was downloaded from Phytozome (http://www.phytozome.net), a web-based platform for green plant comparative genomics (Goodstein et al., 2012).

**Figure 1:**
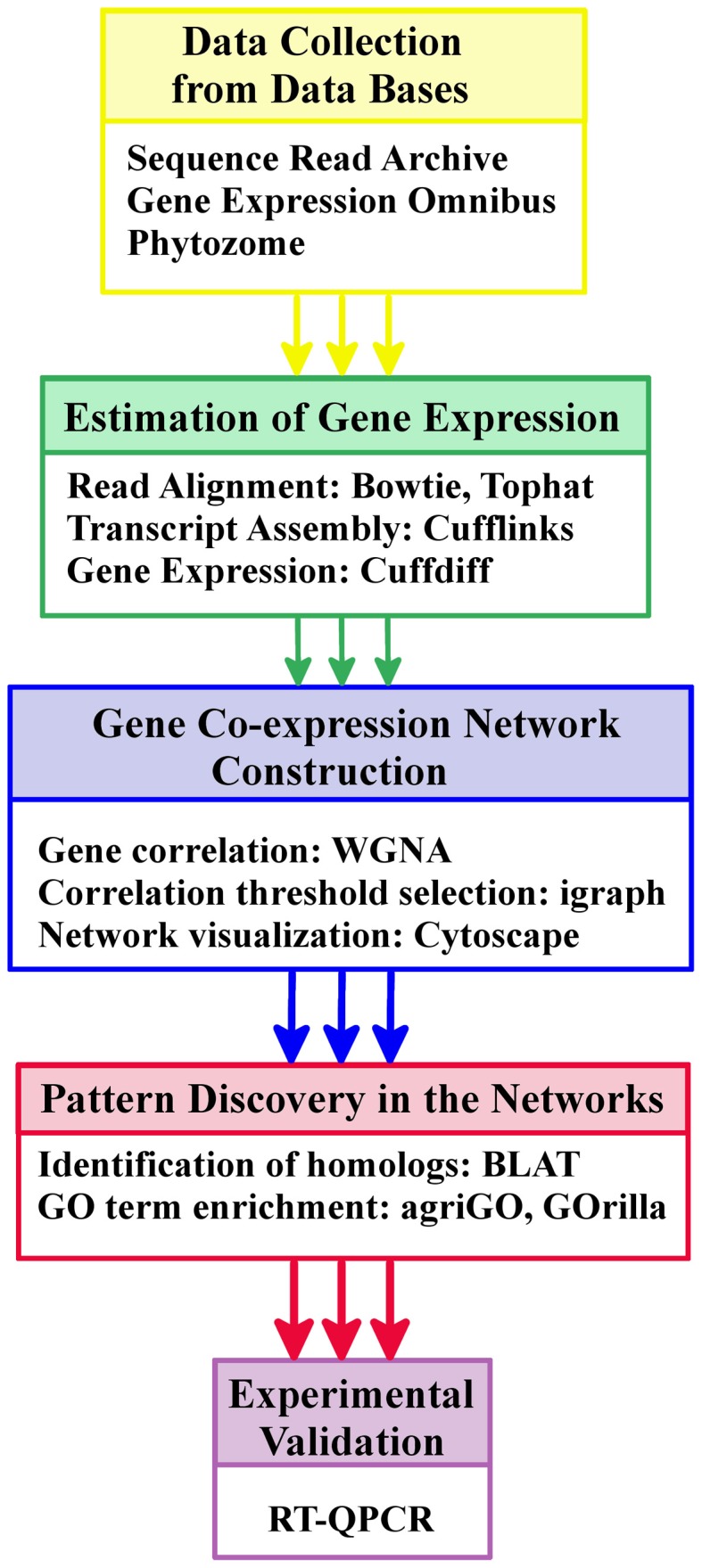
**Schematic workflow including data collection and processing as well as gene co-expression network construction, analysis and validation**. The figure simplifies the main steps followed in the workflow for the construction and analysis of the co-expression networks for *Chlamydomonas*, *Physcomitrella*, and *Arabidopsis*. This workflow requires a reference genome and the corresponding Gene Ontology annotation for each species. The annotation was obtained from the Phytozome database. Gene expression estimation measured in FPKM was obtained by aligning short reads to the reference genome and assembling transcripts using *Tophat* and *Cufflinks*. Gene correlation and network construction were performed with different *Bioconductor* R packages. *Cytoscape* was used for network visualization. Functional patterns in the neighborhood of the genes of interest were identified with the web tools *agriGO* and *GOrilla*. Finally, Q-PCR was used for experimental validation. A detailed representation of this workflow can be seen in Figure S1.

The workflow followed in the RNA-seq data processing is sketched in Figure 1 and detailed in Figure S1. The first step consisted on the alignment of the short read sequences stored in fastq files to the corresponding reference genome using the software package Tophat (Trapnell et al., 2009) to produce BAM files (binary alignment maps). In the second step these files together with the gene annotation information available were used to assemble the whole transcriptomes of *Chlamydomonas* and *Physcomitrella* under the conditions studied. The assembled transcriptomes were stored in GTF files and resolved using the software package Cufflinks and its program Cuffmerge (Trapnell, 2010). Finally, gene expression levels from the different conditions integrated in our study were estimated using Cuffdiff, a program included in the Cufflinks package that takes as input the alignments files (BAM files) and the assembled whole transcriptomes (GTF files). In order to avoid biases due to the length of the different transcripts and number of reads generated in each experiment, Cufflinks estimates gene expression using as unit of measurement the Fragments Per Kb of exon per Million mapped reads (FPKM) (Mortazavi et al., 2008). For the rest of the analysis, exploration, manipulation and visualization of the data generated by Cufflinks we used the R package cummeRbund (Goff et al., 2011).

The analysis of the affymetrix ath1-121501 microarray data obtained for *Arabidopsis* was performed following the methodology described in Hahne et al. (2008). The R package affy (Gautier et al., 2004) from the Bioconductor project (Gentleman et al., 2004) was used for quality control, background correction, normalization with the RMA algorithm (Irizarry et al., 2003), and estimation of gene expression levels in the different conditions under study in this work.

## Results and discussion

### Construction of gene co-expression networks for *Chlamydomonas*, *Physcomitrella*, and *Arabidopsis*

In this analysis, significant co-expression patterns among genes in the transcriptomes of *Chlamydomonas*, *Physcomitrella*, and *Arabidopsis* have been determined analysing the massive amount of gene expression data (see Materials and Methods section) that covers a wide variety of physiological conditions and genotypes (Figure 1). These co-expression patterns were represented using three different gene co-expression networks, one for each species considered in this study representing key steps in the evolution of photosynthetic organisms.

First, in order to remove noise, only those genes that exhibited significant changes in at least one comparison between a condition and its corresponding control, were selected from the studied transcriptomes. This was performed according to the standard approach used for microarray data and its adaptation to the analysis of RNA-seq data (Bullard et al., 2010). The logarithm of the gene expression level measured in FPKM was computed and, using the delta method, the variance of the log odds estimated. *Differentially expressed genes* were then selected combining this information with a fold-change criterion of two in the expression level with respect to the corresponding control.

Next, the expression profiles of the differentially expressed genes were extracted from the gene expression data generated according to the workflow presented in Materials and Methods section. The absolute value of the *Pearson correlation coefficient* between gene expression profiles was used as a measurement of the level of co-expression between the corresponding genes.

Finally, it was necessary to establish a correlation threshold above which it was assumed that two genes are significantly co-expressed. For the rational selection of a gene correlation threshold a criterion that seeks the generation of a scale-free network with a high density was used. This criterion was chosen since most biological networks characterized so far exhibit this scale-free property (Barabasi and Albert, 1999) and because a high-density network facilitates the identification of patterns in the neighborhood of genes (Aoki et al., 2007). For each species, the correlation value for which the *R*^2^ of the linear regression for the logarithmic transform of the node degree distribution presented a maximum while keeping an average of ~20 neighbors per gene was determined. For *Chlamydomonas reinhardtii* the correlation threshold was 0.90, producing a *R*^2^ of 0.9274 and a *p*-value of 3.397e-08 for the scale-free property. For *Physcomitrella patens* the correlation threshold was 0.94 producing a *R*^2^ of 0.9333 and a *p*-value of 8.242e-08 for the scale-free property. For *Arabidopsis thaliana* the correlation threshold was 0.90, producing a *R*^2^ of 0.7827 and a *p*-value of 2.964e-04 for the scale-free property.

**Figure 2:**
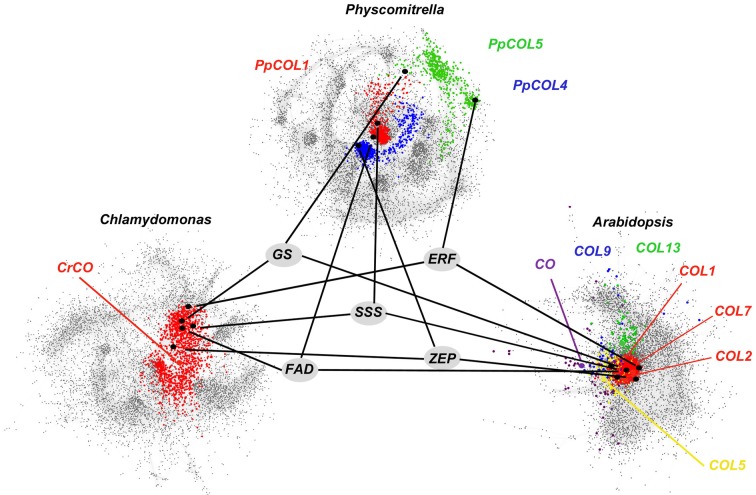
**Gene co-expression networks for *Chlamydomonas*, *Physcomitrella*, and *Arabidopsis***. In the networks, nodes represent genes and edges between nodes show significant co-expression relation between them. *CrCO*, *PpCOLs*, and *AtCOLs* and their neighborhoods (genes three edges apart) are highlighted with different colors. In the *Chlamydomonas* network (bottom left) *CrCO* and its neighborhood (red color) occupy a central position suggesting its role as an essential regulator in the *Chlamydomonas* transcriptome. In the *Physcomitrella* network (top center) *PpCOL1*, *PpCOL4* and their neighborhoods (red and blue colors, respectively) occupy a central position, while *PpCOL5* (green color) is located in the periphery of the network. In the *Arabidopsis* network (bottom right) *AtCOLs* genes (different colors) are widely spread over the network indicating their role in multiple biological processes. Functional conservation, specialization and divergence during the evolutionary line of *COLs* can be observed in their neighborhood. Consistently, *CrCO*, *PpCOLs*, and *AtCOLs* genes are co-expressed with genes involved in light response (*ZEP, Zeaxanthin epoxidase*), starch metabolism (*SSS, solubale starch synthase*), nitrogen metabolism (*GS, cytosolic glutamine synthase*) and response to chemical stimulus (*ERF, ethylene response factor*) as it is suggested by their nearby localization in the corresponding networks.

According to the above methodology three co-expression networks in which edges between genes represent significant co-expression patterns in the analysed conditions were generated. The gene co-expression network for *Chlamydomonas* consisted of 8443 genes and 138,575 significant co-expression relationships. For *Physcomitrella* the corresponding network was constituted by 9080 genes connected by 518,209 significant co-expression relationships. Finally, the gene co-expression network for *Arabidopsis* represented 6204 genes and their 665,034 co-expression relationships. These networks were imported into the software package Cytoscape (Smoot et al., 2011) for their visualization using the organic layout (Figure 2). The corresponding Cytoscape files containing the specification of each network can be downloaded from the link http://ackermann.cs.us.es/web_network/chlamy_physco_arabidopsis.zip for further exploration and analysis.

### In the evolutionary history of the *CO-like* gene family from *Chlamydomonas* to *Arabidopsis* both essential and secondary genes can be identified

The location of genes in co-expression networks and the size of their nearby neighborhood can be used to determine their relevance in the entire transcriptome of the corresponding organism in the conditions under study (Aoki et al., 2007). The three gene co-expression networks analysed in this work are scale-free networks. In this type of networks most nodes are connected with few others. Nevertheless, there exists a small set of nodes that are connected to a large number. These nodes are called hubs (Kleinberg, 1999) and play a key role in the functioning and information propagation in the corresponding networks (Barabasi and Albert, 1999). The hubs in each network were determined using the R package *igraph* (Csardi and Nepusz, 2006). Only genes with a score in the top 5% were considered hubs. The same criterion was used for the three networks. Scale-free networks are robust against random perturbations since these are most likely to hit a node with only a few neighbors and therefore to disrupt only a small portion of the network. Nevertheless, scale-free networks are fragile against perturbations affecting the hub nodes. Therefore, the mutation of a hub node affects a significant number of other nodes connected to it producing a cascade phenomenon that reaches a large part of the entire network (Wang and Chen, 2003). Employing this criterion we can identify central and essential genes as well as peripheral and secondary ones in the evolutionary line from the single gene *CrCO* in *Chlamydomonas* to the 17 *AtCOL* homologues in *Arabidopsis* (Figure 2 and Figure S2).

In the *Chlamydomonas* gene co-expression network, *CrCO* appears located near the core of the network as a hub gene with more than 50 neighbors (Figure 2). This suggests that *CrCO* is an essential gene in the *Chlamydomonas* transcriptome under the conditions studied in this work. A *CrCO* overexpression or mutation would predictably produce a disruption in the functioning of all these neighboring genes that would propagate quickly throughout the network affecting a large number of other genes and the biological processes they are involved in. Therefore, any major change in the expression of *CrCO* could result in an extensive change in the *Chlamydomonas* phenotype. This effect has been reported previously (Serrano et al., 2009) where silencing of *CrCO* using antisense RNA was shown to be detrimental for algal growth. Moreover, overexpression of *CrCO* was reported to produce massive changes in cellular morphology, chlorophyll content, growth rate and cell-cycle regulation.

Using sequence similarity programs from the Phytozome web portal, ten genes were identified in the *Physcomitrella* genome exhibiting high similarity (>40%) with the *Arabidopsis CO* gene (At5g15840) or the *Chlamydomonas CrCO* gene (g6302). These genes that contained at least one B-box and CCT domain, are identified in the current annotation version v1.6 of the *Physcomitrella* genome draft (Table 2). Three of these genes have been studied previously (Shimizu et al., 2004; Zobell et al., 2005) and named *PpCOL1*, *PpCOL2*, and *PpCOL3* respectively. Based on the phylogeny and following the same nomenclature, the rest of the genes were named *PpCOL4* to *PpCOL10*. Only three of these genes *PpCOL1*, *PpCOL4*, and *PpCOL5* are present in the *Physcomitrella* gene co-expression network generated here. The rest of the genes either were not expressed in the samples considered in this study or did not exhibit significant co-expression levels with any other gene in the network. This suggests that these genes play their main role in other physiological conditions not studied in this work. *PpCOL1* and *PpCOL4* are found at the center of the network and although they cannot be regarded as hubs they are connected to other genes that are co-expressed with more than 300 genes. Both genes are placed close to each other suggesting that they may be involved in similar or related biological processes. This would confer redundancy and robustness to the network, so that the alteration in the expression pattern of one of these two genes could be counteracted by the normal function of the other. In this respect, the apparent multiple gene duplication of *CrCO* that gave rise to the ten-gene family of *PpCOLs* has produced redundant hub genes offering robustness to the transcriptome of *Physcomitrella*. The *CrCO* descendant gene in the *Physcomitrella* network, *PpCOL5*, appears in the periphery of the gene co-expression network acting as a hub with 122 neighboring genes. Therefore, *PpCOL5* seems to be a key gene involved in secondary biological processes in this *Physcomitrella* transcriptome since the genes of this cluster are somehow disconnected from the rest of the network (Figure 2). This may suggest that, although a major modification of the expression of *PpCOL5* would not be lethal, it could massively disrupt the biological processes in which it is involved.

Finally, in the *Arabidopsis* gene co-expression network, seven *AtCOL* genes out of a 17-member gene family (Table 2) have been identified. These are *CO*, *COL1*, *COL2*, *COL5*, *COL7*, *COL9*, and *COL13*. The other *AtCOL* genes were not significantly expressed in the conditions considered in this study possibly because they are involved in biological processes not studied here. These *AtCOLs* appear spread all over the network, suggesting that they are involved in a large number of biological processes covering an important part of the transcriptome. *COL1*, *COL2*, and *COL7* are closely arranged at the core of the network (red cluster in Figure 2). In fact, *COL1* and *COL2* are directly connected. The three of them have more than 150 neighbors each and could independently be considered hubs playing a key role in the function and transmission of information in the core of the network. Since these genes are located near each other they are probably involved in related biological processes. A major disruption in the expression pattern of one of these three genes could be easily compensated by the correct functioning of the other two. In fact, single mutations in either *COL1* or *COL2* show no significant effect in the plant (Ledger et al., 2001). Therefore, it could be predicted that, in order to observe any appreciable change in the phenotype a multiple mutant should be generated. This seems to indicate that the new events of gene duplication that took place between *Physcomitrella* and *Arabidopsis* have increased the number of redundant hub genes and, as a consequence, the robustness against external perturbations of its transcriptome. The rest of the *AtCOLs* in our network are distributed toward the periphery and present fewer neighboring genes. It could be inferred then that these genes are not essential hubs and could be involved in secondary processes. For example, a mutation in *CO* limits its effects to the capacity of the plant to flower in response to photoperiod (Rédei, 1962) and its overexpression results mainly in an early flowering phenotype (Putterill et al., 1995). Secondary phenotypic effects not observed in the mutant could arise due to CO overexpression assuming the function of other *AtCOLs*. In a similar situation, *COL5* and *COL9* are located nearby *CO* and their overexpression have been reported to produce a mild early flowering and late flowering phenotypes, respectively (Cheng and Wang, 2005; Hassidim et al., 2009). For *COL13* its situation in the network is analogous but the effect of its mutation or overexpression has not been studied. A similar flowering phenotype could be predicted for this gene when overexpressed.

### The gene neighborhoods of *CrCO*, *PpCOLs*, and *AtCOLs* reveal conservation of co-expressed biological processes across the plant evolutionary lineage

In order to study the different biological processes associated to the genes that constitute the evolutionary line from the single copy *CrCO* in *Chlamydomonas* to the 17 *AtCOLs* in *Arabidopsis* (Figure S2), their neighborhood in the corresponding networks was characterized. For this reason, genes at a distance of at least three, that is, those genes that can be reached using paths with three edges from the genes of interest, were selected. Next, GO (The Gene Ontology Consortium, 2000) term enrichment over the annotation of these genes using the web-based software tools GOrilla (Eden et al., 2009) and AgriGO (Du et al., 2010) was performed, taking the entire genome as background. In the case of *Arabidopsis* we were able to use directly the GO annotation information available at the resource TAIR v.10. Nevertheless, for *Physcomitrella* and *Chlamydomonas* such information is not available due to the lack of previous experimental studies. Instead, for these species, GO annotation inferred from the information in Phytozome and Pfam (Punta et al., 2012) about the protein families to which each gene-encoded protein belongs was used.

First, for the GO enrichment in each one of the three networks, the union of the neighborhood of the corresponding *COL* genes under study, was considered. More specifically, for *Chlamydomonas*, *Physcomitrella*, and *Arabidopsis* the set of genes used to perform GO enrichment were the neighbors at a distance of three of *CrCO*, any of the *PpCOLs* and any of the *AtCOLs*, respectively. At a first glance, a high overlapping between the significantly enriched GO terms in the previous three sets of neighboring genes could be observed. This indicates that the different biological processes in which *CrCO*, some *PpCOLs* and *AtCOLs* are involved are largely conserved across the species evolution. Additionally, new biological processes appeared in the enrichment of the corresponding sets of genes in *Physcomitrella* and *Arabidopsis* that both species share in common (Table 3). This provides evidences of newly acquired influence over different processes in *Physcomitrella* that are afterwards conserved and expanded in *Arabidopsis*.

One of the most significantly enriched GO terms in the regions under study in the three networks from *Chlamydomonas* to *Arabidopsis* is *Response to light stimulus*. Genes located in the neighborhood of *CrCO*, *PpCOLs*, and *AtCOLs* significantly include genes involved in photoperception such as *Cryptochromes* (*CRY*) (*Cre06.g295200*, *Pp1s488_10V6*, *At4g08920*), in redox processes regulated by light such as *Zeaxanthin epoxidase* (*ZEP*) (*Cre02.g095750*, *Pp1s321_9V6*, *At5g67030*) and *Cytochrome P450* (*CYP*) (*Cre07.g325000*, *Pp1s281_82V6*, *At5g05690*) and in the protection against high light intensity such as *Light Harvesting Complexes* (*LHC*) (*Cre17.g740950*, *Pp1s628_3V6*, *At4g10340*) of the photosystem II. This response to light has been previously reported for *CrCO* (Serrano et al., 2009), *PpCOL1* (Zobell et al., 2005) and *CO* (Valverde et al., 2004). Therefore, this interspecies network analysis could successfully predict gene function using the significantly enriched GO terms observed in the corresponding modules around the genes of interest.

The next conserved GO terms are involved in metabolism such as *Starch metabolic process; Lipid metabolic process* and *Nitrogen compound metabolic process*. Consistently, in the regions of the three networks, genes involved in starch biosynthesis such as *Starch Synthases* (*SSS*) (*Cre16.g665800*, *Pp1s234_74V6*, *At4g18240*), in starch hydrolysis such as *Beta-amylases* (*BAM*) (*Cre01.g044100*, *Pp1s317_42V6*, *At2g32290*), in fatty acid synthesis such as *Long Chain Acyl-CoA synthases* (*LACS*) (*Pp1s113_124V6*, *At2g47240*) and *3-ketoacyl-CoA synthase* (*KCS*) (Cre07.g320550, Pp1s268_29V6, At4g34250), in fatty acid modification such as *Fatty Acid Desaturases* (*FAD*) (*Cre13.g590500*, *Pp1s98_209V6*, *At3g15850*), *Glutamine Synthetase* (*GS*) (*g13061*, *Pp1s19_281V6*, *At5g37600*) and *Nitrate Transporter* (*NTR*) (*g18260*, *Pp1s283_88V6*, *At4g18480*) can be found. Recently, the influence of *CrCO* and *CO* over the metabolism of starch and carbohydrates has been described (Serrano et al., 2009; Ortiz et al., unpublished results). The effect of these genes over the metabolism of lipids and nitrogen compounds suggested in this work remains to be explored experimentally. This reflects the possible application of this type of analysis, employing the evolutionary line of gene co-expression networks, for gene function prediction. This way, when a gene with an unknown function is consistently co-expressed across its evolutionary lineage with the same well-characterized genes, a putative function could be assigned to it.

Finally, several GO terms defined as *Response to chemical stimulus* and *Developmental process*, were identified that significantly and specifically appeared enriched both in the *Physcomitrella* and *Arabidopsis* networks. Although these GO term did not appear significantly represented in the neighborhood of *CrCO* in the *Chlamydomonas* network we were still able to identify several algal genes that showed a similar annotation, including *Ethylene Response Factors* (*ERF*) (*g15714*, *Pps265_50V6*, *At1g53910*) and *Auxin Transporters* (*AUX*) (*Cre16.g680200*, *Pp1s167_10V6*, *At5g43700*). Other GO term such as *Response to jasmonate* only presented conserved genes in *Physcomitrella* and *Arabidopsis* networks such as *Jasmonate Insensitive* (*JIN*) (*Pp1s11_350V6*, *At1g32640*) and *Jasmonate Responsive* (*JR*) (*Pp1s200_12V6*, *At3g16470*) genes. In fact, it has been suggested that *Chlamydomonas* can respond to some hormonal stimuli and that this effect has stretched and become more complex as plants developed intricate structures and functions, reaching the complexity of the current plant hormone responses (Riaño-Pachon et al., 2008). These could be identified as the precursors of genes associated to hormone signaling and experiments could be designed to demonstrate their function. Therefore, another application of this cross species network analysis could be to predict the evolution of physiological processes and their importance in the diversification of key regulatory responses (such as hormone responses) between different species. If this is the case, when applied to a species transcriptome, implying the study of regulatory modules of many key factors in a natural population context, this analysis could be a very useful tool to understand speciation.

### Gene specialization and the establishment of connections between different biological processes is reflected in the evolutionary history from *CrCO* to *AtCOLs*

Previously it was shown that, in the multi-gene families of *Physcomitrella* and *Arabidopsis*, different genes appear at distant positions in the network showing distinct levels of importance in the organization and information transmission of the corresponding networks. In order to determine if this influences the biological functions of genes, GO term enrichment in the neighborhood of each individual gene of interest in this work, was performed.

**Figure 3:**
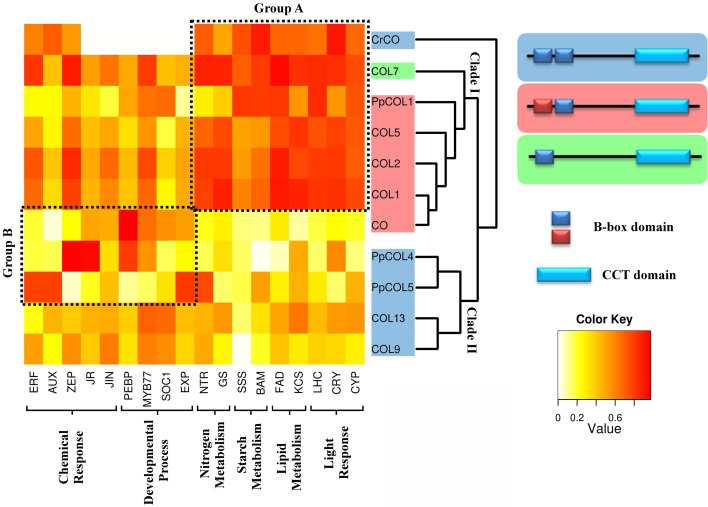
**Comparison of the gene co-expression analysis with a phylogenetic and domain conservation study**. The analyzed CrCO, PpCOLs, and AtCOLs proteins are organized in the rows of a heatmap according to their phylogeny. Two big clades descending from CrCO can be observed. Clade I includes genes with a divergent and a normal B-box (PpCOL1, COL5, COL2, COL1, and CO), highlighted in red, and COL7 with a single B-Box. Clade II is composed of proteins with two similar B-boxes (PpCOL4, PpCOL5, COL13, and COL9). Representative clustered genes in biological processes in which COLs are involved were chosen. Dark red cells indicate a high correlation between corresponding genes, while white cell indicate absence of correlation. Group *A* have retained CrCO ancestral functions related to light response and carbon metabolism (PpCOL1, COL7, COL5, COL2, and COL1). Group *B* is significantly correlated with chemical response and developmental processes (CO, PpCOL4, and PpCOL5). Some connection between groups is still observed. Functional classification coincides mostly with phylogeny with exceptions like COL1 and CO, phylogenetically very close but functionally very different. In contrast, CO, PpCOL4, and PpCOL5 are very divergent phylogenetically but seem to have functionally converged.

In the *Chlamydomonas* genome a single *CrCO* copy involved in core biological processes can be identified: response to light stimulus, starch metabolic process, lipid metabolic process and nitrogen compound metabolic process. Attending to our analysis *CrCO* could also be marginally involved in pre-developmental processes and response to chemical stimuli (Table 4 and Figure 3).

**Table 4:**
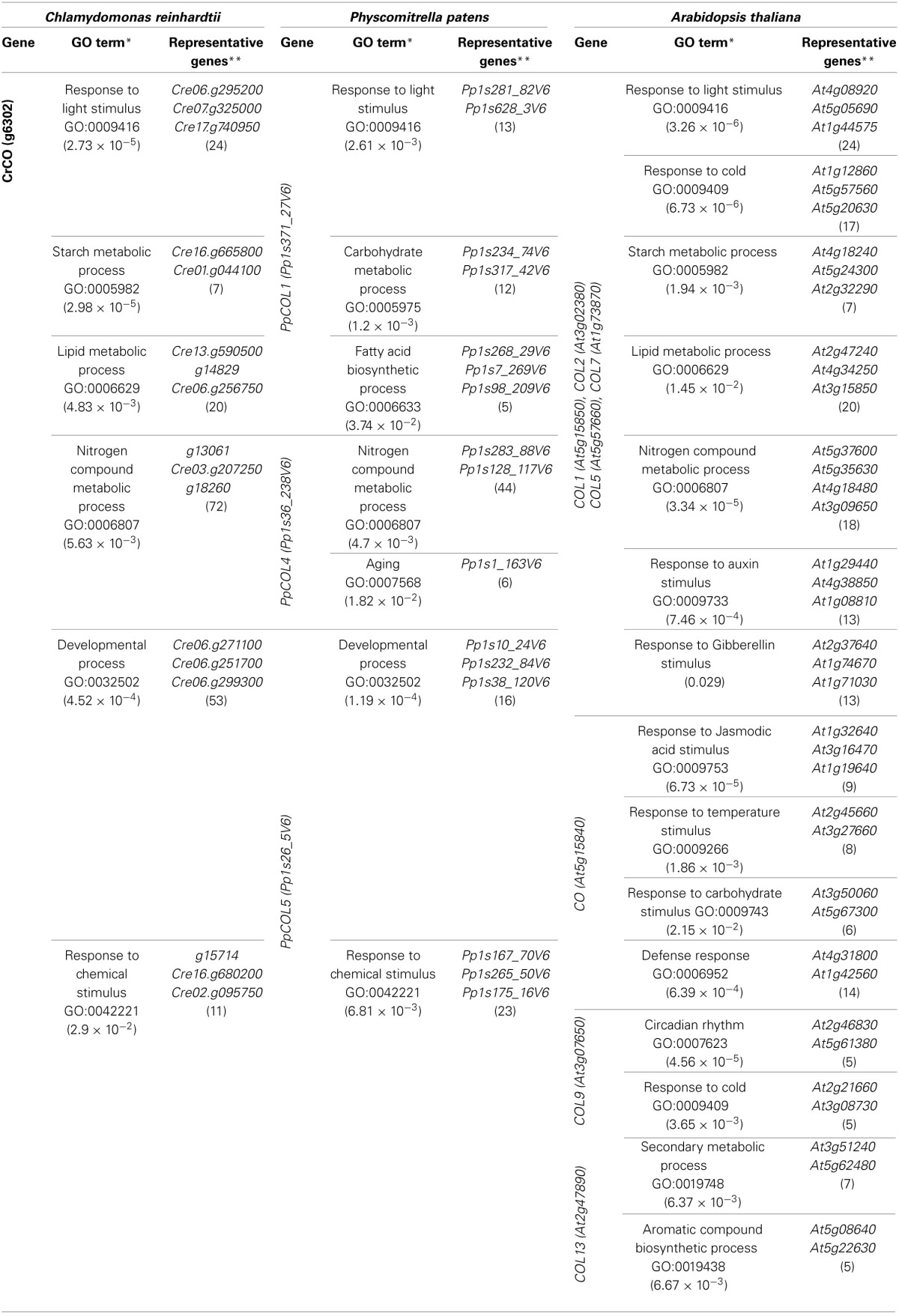
**GO terms significantly enriched in *CrCO*, *PpCOLs*, and *AtCOLs* neighborhood in the gene co-expression networks**.

For each species, Chlamydomonas, Physcomitrella and Arabidopsis, the COL genes that appear in the networks are associated with the GO terms that are significantly enriched in their neighborhoods (^*^). The p-values are shown between parentheses below each GO term. For each GO term significantly enriched in the corresponding neighborhood, several representative genes are listed using the identifiers from Phytozome and TAIR databases (^**^). The total number of genes identified for each GO category is shown below between parentheses.

In the *Physcomitrella* network three *COL* genes were identified: *PpCOL1*, *PpCOL4*, and *PpCOL5*. GO term enrichment in each of the neighborhood of these genes show that each one has diverged to specialize in a particular biological process. *PpCOL1* exhibits a high level of co-expression with LHC, SSS and fatty acid denaturizes. This suggests a specialization of this gene in response to light and carbon metabolism, consolidating a connection that was already established in *Chlamydomonas*. On the other hand, in the neighborhood of *PpCOL4* and *PpCOL5*, genes associated with nitrogen metabolism, aging, development and response to chemical stimuli can be found. This suggests that these processes, that were secondary to *CrCO* in *Chlamydomonas*, became related to new *COL* genes and possibly more relevant during the evolution of plants into species like *Physcomitrella*. Duplication and diversification from *CrCO* produced genes such as *PpCOL4* and *PpCOL5* that covered the new challenges related to maintaining a multicellular organism in order to coordinate, among others, its developmental processes. Of special interest among the genes highly co-expressed with these two genes is the *Physcomythrella PEBP, Phosphatidyl-Etanolamine Binding Protein (Pp1s32_140V6)*. This gene shows a high sequence similarity with the *FT* gene, the mobile florigen hormone that constitutes the main target of *CO* in *Arabidopsis*. Therefore, another possible application of our approach would be to identify true functional orthologous genes by determining whether or not their neighboring genes of known functions in the corresponding networks are evolutionarily conserved.

In the *Arabidopsis* network, a considerable increase in the complexity and diversification of the function of the seven *AtCOL* genes included in this study is apparent. A group of genes comprising *COL1*, *COL2*, *COL5*, and *COL7* are co-expressed with genes whose function is similar to the core processes associated with *CrCO*, such as response to light, starch, lipid and nitrogen metabolism. Additionally, in *Arabidopsis* these genes show a connection with biological processes not annotated before such as response to cold, auxin and gibberellin that have been previously described as major regulators of plant development. This may suggest that the level of regulation exerted by these *AtCOL* genes is more complex than the one exerted by their ancestral genes *CrCO* and *PpCOL1*. This suggests that in *Arabidopsis*, besides a light input, the *AtCOLs* associated with carbon and nitrogen metabolism also receive inputs from temperature and hormone signaling. The key gene in the *Arabidopsis* photoperiodic response *CO*, is co-expressed with genes associated with responses to jasmonic acid, temperature stimuli, carbohydrate stimuli and defence to pathogens. This could provide evidence for yet unexplored effectors that trigger the floral transition in *Arabidopsis* associated to *CO* function. *COL9* seems to be significantly influenced by circadian rhythms and response to cold whereas *COL13* is associated with very specific processes such as aromatic compound biosynthesis. Therefore, these co-expression networks could be used to predict complex functional links within species based on their positioning and their relation to primitive functions in ancestral species.

In order to determine the degree of co-expression and specialization between the genes of interest and the different biological processes discussed above, the level of correlation between them and some representative genes for each biological process was plotted (Figure 3). This plot separates two distinct groups of genes. Group A is constituted by *CrCO*, *PpCOL1*, *COL5*, *COL7*, *COL2*, and *COL1*. It shows a high correlation with the core biological processes covering response to light (*CYP*, *CRY*, *LHC*), lipid (*FAD*, *KCS*), starch (*SSS*, *BAM*), and nitrogen (*NTR*, *GS*) metabolisms. These, rather than developmental processes, could then be appropriately considered as the ancestral main function of *CrCO* and as such these functions have been conserved across the plant evolutionary line for all *COLs*. Group B comprises *CO*, *PpCOL4*, and *PpCOL5*. These genes are highly co-expressed with previously described genes associated to developmental processes [*PEBP; MYB77; Suppressor of Overexpression of CO 1*, (*SOC1*); *expansin*, (*EXP*)] but also interestingly with the response to chemical stimuli (*ERF*, *AUX*, *JR*, *JIN*). Finally, *COL9* and *COL13* seem to be equally correlated with all the different biological processes discussed before.

Although, the genes from group A (Figure 3) are highly co-expressed with light response and metabolic processes they also show a considerable correlation with genes involved in development. This could hint to a role of this group of *COLs* in developmental regulation or that they may receive inputs from developmental processes in order to exert a tighter control on metabolism. Similarly, group B genes, for example *CO*, in spite of being significantly co-expressed with genes involved in developmental processes and response to hormones, also shows an appreciable degree of correlation with genes involved in metabolism. Therefore, these genes may receive inputs from metabolic processes in order to produce a more effective control over developmental processes or they may also regulate, up to some degree, carbon and nitrogen metabolism. In any case, these two groups of genes seem to effectively establish connections between development, metabolism and light response through the *PpCOLs* and *AtCOLs* that evolved from the core connections established by *CrCO*. To demonstrate this hypothesis, these interconnections will need to be further explored and validated experimentally. Some late results from our laboratory suggest that indeed control of sugar release during the floral transition in *Arabidopsis* depends on photoperiod. This control is exerted through transcriptional regulation of a starch synthase gene by *CO* (Ortiz et al., unpublished data). This functional connectivity between hubs and functions may be in the base of the complexity found in the response of plants (and any other organism) to stimuli. By naturally conditioning and constraining gene function within these functional modules, genes cannot evolve individually; they retain part of the regulation of their ancestors and therefore involve complexity at the same time that they promote their joint regulation. This would be explained in more detail further on.

### Functional studies based on sequence similarity are not sufficient to explain the evolutionary history of the *COL* genes

As described above, the integration of transcriptional information and the identification of gene expression patterns in the gene co-expression networks suggest a specific classification of the *CrCO*, *PpCOLs*, and *AtCOLs* according to their associated biological processes. It was interesting to explore whether or not this was in agreement with more classical approaches based on sequence similarity such as phylogenetic analysis and identification of conserved protein domains.

CrCO, PpCOLs, and AtCOLs belong to the COL protein transcription factor family, which is characterized by the presence of a specific set of conserved domains. All of them contain a CCT domain in the C-terminal part that has been shown to be involved in nuclear import (Robson et al., 2001), DNA binding activity (Tiwari et al., 2010) and in protein-protein interactions such as the binding of CO to the ubiquitin ligase COP1 (Jang et al., 2008). In the N-terminal part, COLs present one or two specific Zinc-finger domains called B-boxes with protein-protein interaction function (Khanna et al., 2009). In the subfamily of AtCOLs studied here, only COL7 has one single B-box. According to these B-boxes we can classify the CrCO, PpCOLs, and AtCOLs proteins depending on the divergence degree of the second B-box. CrCO, PpCOL4, PpCOL5, COL9, and COL13 proteins have two almost identical B-boxes, whereas in PpCOL1, COL5, COL2, COL1, and CO, the amino terminal B-box has diverged (Figure 3 and Figure S2).

Phylogenetic analysis of the CrCO, PpCOLs, and AtCOLs has provided insights into their evolutionary history (Griffiths et al., 2003; Zobell et al., 2005; Valverde, 2011). In this study, the genetic distances between the different COLs found in the networks were determined and a phylogenetic tree was built (Figure 3). In this phylogenetic tree, two clades descending from the ancestral CrCO protein could be distinguished. Clade I comprises PpCOL1, COL7, COL5, COL2, COL1, and CO, whereas PpCOL4, PpCOL5, COL9, and COL13 constitute clade II. These clades are separated by an event of gene duplication that took place before the subsequent event of speciation that gave rise to *Physcomitrella* and *Arabidopsis*. Therefore, these two groups of proteins seem to have long diverged in the plant evolutionary line.

When comparing the clades in the phylogenetic tree with the classification of *CrCO*, *PpCOLs*, and *AtCOLs* based on the biological processes associated with them, it was observed that genes of recent descent do not necessarily show a conserved association with the same biological processes. For instance, *CO* and *COL1* are known to be the result of a very recent event of gene duplication and the proteins share more than 80% of amino acid identity. Nevertheless, *COL1* appears in group A, related to light response and metabolism whereas *CO* is a member of group B involved in developmental processes. This divergence in functionality, in spite of the high sequence identity, has been shown experimentally by the inability of an over-expressor of *COL1* to affect flowering (Putterill et al., 1995). In contrast, although *CO* and *CrCO* are evolutionary more distant, *CrCO* is able to accelerate the floral transition in *Arabidopsis* (Serrano et al., 2009). Similarly, although *PpCOL1* presents a high sequence similarity with *CO* and belongs to the same clade it does not affect the flowering phenotype when over-expressed in *Arabidopsis* (Zobell et al., 2005). According to our classification based on biological processes associated with each gene, *PpCOL4* and *PpCOL5* could be better candidates to affect the flowering process, since they belong to the group, as *CO* does, associated with developmental processes. This could mean that some genes presenting distant evolutionary origins can converge to acquire influence over the same biological processes.

This gene evolution line that combines divergence of functionality in those of recent descent and convergence to the same biological processes of evolutionary distant ones is not unique to *CrCO*, *PpCOLs*, and *AtCOLs*. A similar evolutionary history has been described for the family of GATA transcription factors in *Arabidopsis* (Manfield et al., 2007). In fact, a similar approach to study the phylogeny and function of GATA and DOFs transcription factors employing these co-expression networks gives similar results (data not shown) confirming the applicability of these approaches to other gene families.

Therefore, the analysis of gene co-expression networks presented here provides new insights into gene evolutionary lineage following duplication events. These duplications can result in function divergence from their ancestors such as the case of *PpCOL1*, *COL1*, and *CO* as well as convergence to the same function through different branches of the evolutionary tree, as is the case of *CO*, *PpCOL4*, and *PpCOL5*. Experimental demonstration of an involvement of PpCOL4 or PpCOL5 proteins in developmental processes in *Physcomitrella*, such as is the case of CO in *Arabidopsis*, would strongly support this point.

### Experimental validation supports the predictions inferred using gene co-expression networks

**Figure 4:**
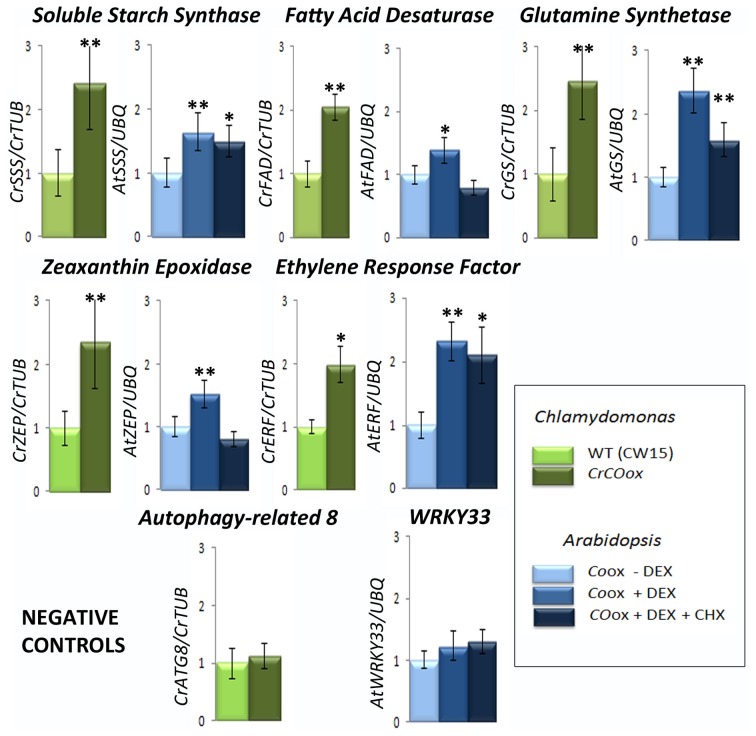
**Genes within the *COL* co-expression network are influenced through evolution by *COL* overexpression**. The bars represent Q-PCR expression levels of some representative genes within the influence of COLs in the networks of Figure 2. The results for gene homologues of Soluble Starch Synthase (*SSS*), Fatty Acid Desaturase (*FAD*), Glutamine Synthetase (*GS*), Zeaxanthin Epoxidase (*ZEP*), and Ethylene Response Factor (*ERF*) in *Chlamydomonas* (in green) and *Arabidopsis* (in blue) are presented. Two control genes, *ATG8* from *Chlamydomonas* and *WRKY33* from *Arabidopsis* that show no correlation with either *CrCO* or any *AtCOL* respectively, were chosen as negative controls. Light green bars represent relative gene expression levels in wild type (*CW15*) *Chlamydomonas* while dark green represent *CrCO*ox algae results. Light blue bars represent relative gene expression levels in 35S:*CO*-GR *Arabidopsis* plants without chemical addition; medium blue bars represent the same in the presence of DEX and dark blue in the presence of DEX and CHX. Data are the mean of 4 independent experiments relative to control genes and include s.e.m. Two asterisks above a bar represent a fold change with 95% significance, while a single asterisk represents a fold change with 90% significance.

In order to asses if the observations about the conservation of function in the evolutionary line of *COL* genes under study were correct, an expression analysis was performed. *Arabidopsis* plants overexpressing *CO* from a 35S promoter and fused to the rat glucocorticoid receptor (GR) were employed to identify CO targets (Simon et al., 1996). In *Chlamydomonas*, an approach employing recombinant alga expressing *CrCO* under the conditional expression of the *pnia2* promoter, which induces gene expression with a change in the nitrogen source (González-Ballester et al., 2005) was performed. In both cases, primers for characteristic genes co-expressed with *CrCO* or in the neighborhood of other *AtCOLs* were designed (Table 2) and their expression tested by Q-PCR (Figure 4).

In *Chlamydomonas CrCO* expression was induced by transferring ammonia-grown cells in LD (when *nia2* promoter is inactive) to nitrate growing conditions, when the *nia2* promoter is activated and produces high amounts of *CrCO* transcript (Serrano et al., 2009). In *Arabidopsis*, 35S:*CO*-GR plants growing in plates in LD were exposed to DEX treatment for 4 h and samples taken (see Materials and Methods section). Addition of CHX (a potent translation inhibitor) in samples assured that gene expression was due to a direct CO induction (Samach et al., 2000). In both experiments, total RNA was extracted, cDNA constructed, and Q-PCR analyzes performed in control and experimental samples at least three times.

In Figure 2 we had shown that genes coding for a Soluble Starch Synthase (*SSS*), a *FAD*, a Glutamine Synthetase (*GS*), a *ZEP* and an *ERF* were positively co-expressed with *CrCO* in the *Chlamydomonas* gene co-expression network. In *Physcomitrella* and *Arabidopsis* this effect was conserved, but distributed among different *COL* clusters, so that i.e., in *Physcomitrella* the *GS* homologue was co-expressed with *PpCOL4* (in blue) while in *Arabidopsis* was in the neighborhood of *AtCOL9*. In the Q-PCR experiments performed in *Chlamydomonas*, as expected by the results of the massive analysis, *CrCO*ox algae presented an increase of 2–3-fold in the mRNA levels of these genes (Figure 4, dark green bars) compared to control levels (light green bars). When the mRNA expression levels for *SSS*, *FAD*, *GS*, *ZEP* and *ERF Arabidopsis* gene homologues in 35S:*CO*-GR plants after DEX treatment were tested (Figure 4, medium blue bars), we could also detect an increase in mRNA levels compared to control (light blue bars). This effect was high for *ERF* and *GS* mRNA levels and very low for *ZEP* and *FAD*. In fact, when plants were subjected to both CHX and DEX treatments (Figure 4, dark blue bars) some of this effect was reduced, particularly for *FAD*, *ZEP* and in a lower extent for *GS*, while mRNA levels remained constantly high for *SSS* and *ERF*. This probably indicates that both *SSS* and *ERF* are *bona fide* targets of *CO*, while the other three, in the case of *Arabidopsis*, are not. This effect could be due by *CO* activating the expression of genes that are true regulators of their expression, or because *CO* can, to some extent, still affect their translation, as in the case of *GS*, where a small increase in transcript levels can be observed after CHX addition. In any case, genes randomly chosen because they were sufficiently far from *CrCO* (*ATG8*) or *AtCOLs* (*WRKY33*) core control cluster in the network, did not show any enhanced expression level in the induction experiments neither in *Chlamydomonas* nor in *Arabidopsis* (Figure 4).

Some of the genes activated by CO in *Arabidopsis* may be direct targets, meaning that CO protein could bind directly to their promoters. If this was the case, the promoters of these genes would be enriched in the CO binding sites described in the literature (CORE and HAP) (Wenkel et al., 2006; Tiwari et al., 2010) when compared to a background of genes that are not co-expressed with CO. In order to corroborate this a transcription factor binding site enrichment analysis was performed using 1000 bp of the promoter region of several genes in the neighborhood of CO, including those analysed by Q-PCR, taking as background genes randomly chosen far from CO in the network. Indeed, we could observe enrichment in the promoter of co-expressed genes with CO for the CORE binding site with a *p*-value for the Fisher's exact test of 0.0027. 70.7% of the genes in the target exhibited at least one CORE site in their promoter whereas only 20.5% of those far from CO presented at least one CORE site (Figure S3). Therefore, when studying transcription factors in gene co-expression networks, a transcription factor binding site enrichment analysis over the promoters of genes in their neighborhood may also shed light over the binding site of the transcription factor under study and the kind of regulation exerted (direct or indirect) over them. The transcription factor binding site enrichment analysis was performed using the software package HOMER (Hypergeometric Optimization of Motif EnRichment) (Heinz et al., 2010).

This and previous gene expression analyzes were supporting two clear ideas. In the one hand, the network of genes influenced by *COLs* is conserved between two extremely distant organisms such as the chlorophyte *Chlamydomonas* and the brassica *Arabidopsis*. Second, it was also indicating that during evolution, some kind of diversification had occurred in the *COL* gene family so that different *COLs* had specialized in different functions (different gene co-expression clusters). In fact, when *CO* is overexpressed in *Arabidopsis*, its main target *FT* augments its levels of expression more than a 100-fold (Valverde et al., 2004), much more than any of the targets tested here. When, on the other hand *CrCO* is overexpressed in *Arabidopsis*, an induction of *FT* expression can also be observed, but never reaching the levels of CO overexpressing lines (Serrano et al., 2009). Therefore, when an experiment designed to observe the function of a transcription factor within a gene family is planned, gene co-expression networks, employing this evolutionary perspective, could provide an advantage in order to identify the correct gene targets and the functions they are involved in.

### The evolutionary history of *CrCO*, *PpCOLs*, and *AtCOLs* can be explained by the innovation-amplification-divergence model of evolution by gene duplication

Gene duplication was proposed as one of the major forces driving the evolution of genes (Ohno, 1970; Kimura and Ohta, 1974) in the early 1970s. Nevertheless, the initial theory of evolution by gene duplication, *Mutation During Non-functionality*, presented several problems. One of the most important ones is called Ohno's dilemma. The duplication of a gene increases the metabolic load of the organism producing an evolutionary disadvantage. This will remove the new copy of the gene from the population preventing it from staying long enough to accumulate mutations that will confer it a new functionality. In order to overcome these problems several models of evolution by gene duplication has been developed in the last years (Zhang, 2003). Among these, the *Innovation-Amplification-Divergence* model (Bergthorsson et al., 2007) is the one that better fits the evolutionary history of the *CrCO*, *PpCOLs*, and *AtCOLs* genes inferred in this work using gene co-expression networks. Recently, this model of evolution by gene duplication has been proved experimentally in bacterial systems (Nasvall et al., 2012).

**Figure 5:**
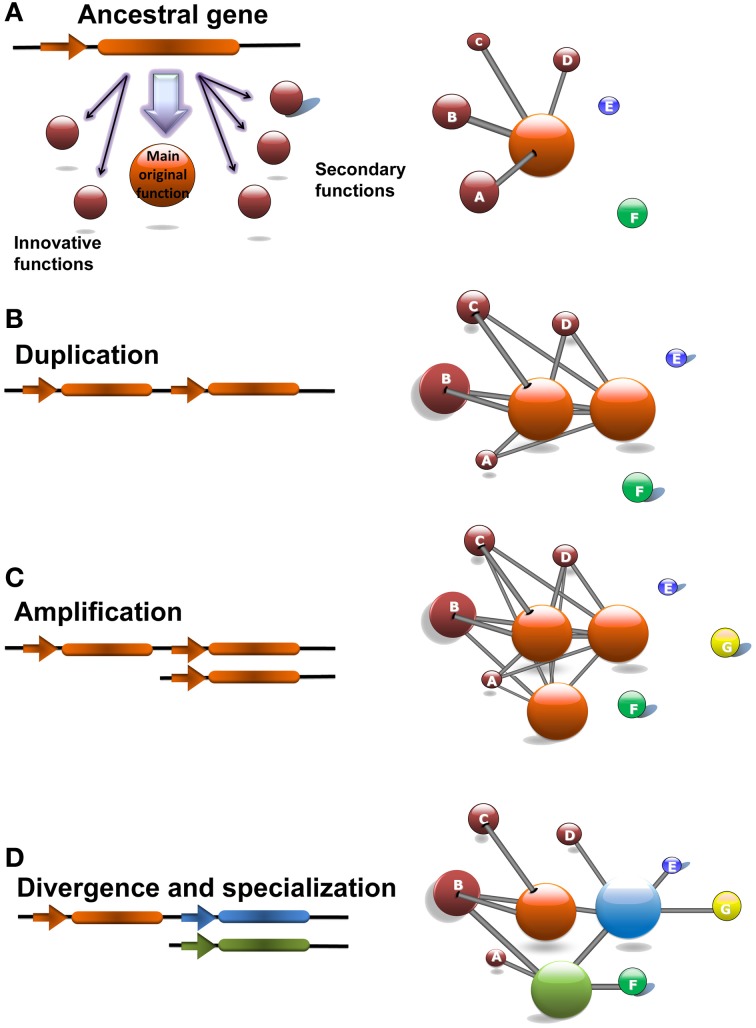
**The Innovation-Amplification-Divergence (IAD) model of evolution by gene duplication explains the evolutionary history of the *CrCO*, *PpCOL*, and *AtCOL* genes. (A)** The *CrCO* gene in *Chlamydomonas* is a hub gene whose main functions are light response and carbon/nitrogen metabolism. Additionally, *CrCO* presents links to secondary functions such as response to chemical stimulus and developmental processes that can be considered innovations developed by this gene. **(B)** During the evolution of multicellular land-based plants such as *Physcomitrella* and *Arabidopsis* these secondary functions became selectively valuable which facilitated the fixation of duplications of *CrCO*. Initially, duplicated genes are identical and conserved exactly the same links as the ancestral gene. **(C)** In order to fully cover the demands exerted by selection of a more complex regulation over developmental processes, an amplification consisting of multiple gene duplications took place, giving rise to the 10 gene family of *PpCOLs* and the 17 gene family of *AtCOLs*. **(D)** After duplication and amplification the same selectively forces facilitate the accumulation of mutations over the duplicated genes that make them diverge, specialize and acquire new functions as it can be observed in the *PpCOL* and *AtCOL* genes. This is reflected in the network by the removal of links and the establishment of new ones. Note that in spite of divergence the genes tend to keep links to the main ancestral functions.

According to this theory, before duplication, the ancestral gene needs to be involved in a wide range of biological processes that include its primary function as well as several secondary processes that would constitute the innovation pose by the gene (Figure 5A). Under these conditions a high copy number of the proteins produced by the gene is needed. Therefore, for such a gene, a duplication that would eventually increase the number of proteins is not as detrimental as for other genes for which a low number of proteins is required (Figure 5B). In this respect, ancestral genes subject to produce a fixable duplication would appear as hubs in gene co-expression networks being involved in a wide range of biological processes through its neighboring genes (Li et al., 2006). Due to a change in the environment, one of these secondary processes may become selectively valuable and would demand an increase in the dosage of the ancestral gene actively fixing its duplication. Since initially the new gene does not efficiently cover the needs of the secondary function of the ancestral gene a series of several duplications, known as the amplification phase, are needed to completely satisfy the new evolutionary requirements (Figure 5C). After duplication the same evolutionary pressure facilitates a divergence phase where mutational improvements over the new genes make them specialize in the new biological processes that pose a selective value (Figure 5D).

In this network analysis, originally the *CrCO* gene that appeared for the first time in the chlorophytes such as *Chlamydomonas* constitutes a hub gene related to a wide variety of biological processes. A single gene copy of *CrCO* would therefore efficiently regulate light response and metabolic functions. Nevertheless, in a marginal and less efficient way it would also be involved in secondary biological functions related to response to chemical stimulus and pre-developmental processes (such as hormonal response). When multicellular photosynthetic organisms such as bryophytes like *Physcomitrella* required a more complex regulation of their developmental processes the secondary functions of *CrCO* became selectively valuable. This event facilitated the positive selection of the duplication of *CrCO* that was fixed in the genome by the new demands posed by complexity (multicellularity, new aerial habitats, intricate developmental processes, …). Since, initially *CrCO* did not efficiently regulate processes related to development, multiple duplications, as the one observed in the *PpCOLS* ten-gene family of the moss, were necessary during the amplification phase to completely cover the developmental needs of multicellular photosynthetic organisms. As the complexity increased during the evolutionary line leading to the flowering plants, more duplications were needed. This gave rise to the *Arabidopsis* 17 *AtCOL* gene family in order to cover all the complex stimuli governing the numerous developmental processes of an angiosperm, such as the flowering transition. After duplication, each new gene was free to diverge and specialize in one or several of the secondary functions and to acquire links to new biological processes. Nevertheless, these new genes always kept a link with the original network around their ancestral gene, thus keeping some of its ancestral functions. This has been shown recently in the case of the role of *CO* gene in *Arabidopsis* linking the photoperiodic floral transition with starch metabolism (Ortiz et al, unpublished results). As the analysis of gene co-expression network unveils, CO, although mainly involved in the flowering transition, still keeps influences over sugar and starch metabolism. This constitutes a connection between carbon metabolism and flowering (Wahl et al., 2013) that needs to be further studied.

## Concluding remarks

The family of *COL* genes that regulate photoperiodic responses in photosynthetic eukaryotes is a particularly suitable family of transcription factors to perform evolutionary studies. In the first hand, it is constituted by a single copy gene in chlorophytes, where it was first originated (Serrano et al., 2009). On the second hand it has become a family of *CO-like* genes during the evolution of complex photosynthetic organisms, with a 10-gene member family in *Physcomitrella* and 17 close members in *Arabidopsis*. This amplification and diversification have allowed for functional specialization while genes still kept links to their original ancestral biological functions.

In our evolutionary analysis, gene co-expression networks establish constrains to the evolution of genes which is particularly evident in those that constitute hubs, nodes that are linked to a large number of genes at the expression level (often, but not only, transcription factors). One can visualize a gene co-expression network as a spider web formed by nodes and edges representing genes and the co-expression relationships between them. Therefore, pulling the string of a gene will drag its co-expressed genes as well. This can be achieved by modifying its expression as drastically as in a null mutation, which will have an effect on the other genes connected to it. The more genes are connected to a particular gene, the more modifications it will create in the network, and the constrain to change will be higher.

Therefore, in our model, genes could not evolve independently but constrained by the limits exerted by the co-expression network in which they are imbibed. For example, gene duplication could not allow the acquisition of new functions randomly, but only within the possibilities already established by the parental gene. This way, genes keep links with ancestral functions while acquiring new ones, thus making the networks more complex and allowing for co-regulation of different functions. This could explain why organisms keep different levels of coordination in different organization levels (such as coordinate metabolism and growth) and acquire complexity while at the same time are able to acquire new functions in order to make more intricate and fitted their responses to external and internal stimuli.

Our results can then explain some previous natural observations on gene evolution while at the same time include a predictive analysis. By studying co-expression networks of a transcription factor throughout the evolutionary life of related organisms we can observe the natural progression of its function and the modifications in the co-expression cluster it is immersed. Observing conserved and diversified correlated networks we could make predictions on what the function of the gene would be in any other organisms and the effect it would have in the physiology of that organism. Thus, for example, employing the correct species and conditions gene co-expression analysis with an evolutionary perspective could be very helpful in agriculture to design plants *a la carte* to confront the challenging biotechnological problems of this century.

### Conflict of interest statement

The authors declare that the research was conducted in the absence of any commercial or financial relationships that could be construed as a potential conflict of interest.
