# Supplementary material for: A contribution to the study of plant development evolution based on gene co-expression networks
Source: Front Plant Sci. 2013 Aug 5;4:291. doi: 10.3389/fpls.2013.00291 (PMC3732916; doi:10.3389/fpls.2013.00291)
Supplement: Supplementary file 1 [file Presentation1.PDF]

Supplementary Figures.

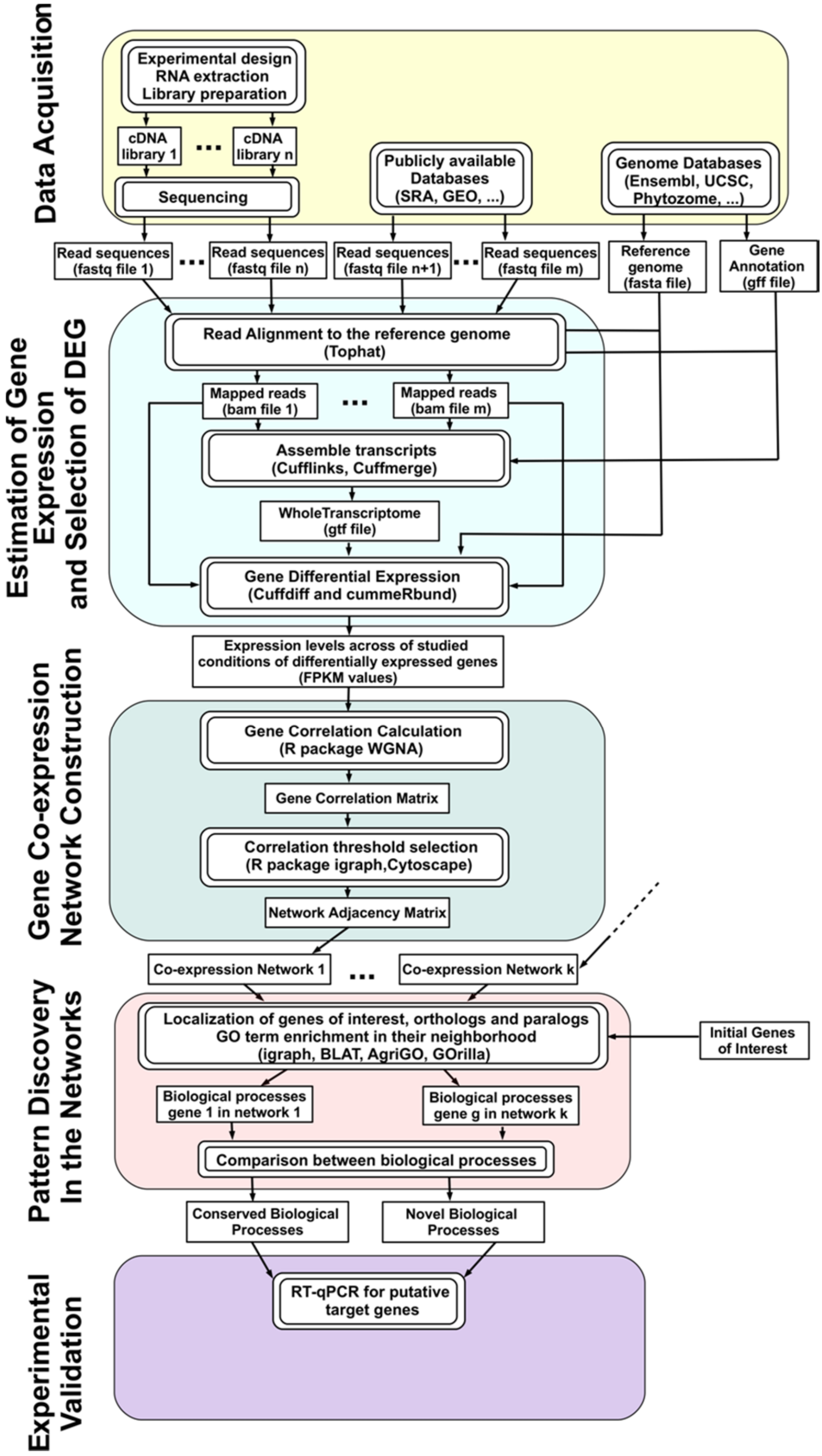

Figure S1. Workflow for gene co-expression network construction and analysis. The workflow used in this study relies on the availability of a reference genome and Gene Ontology (GO) annotation. These data were downloaded from the genome databases *Phytozome* and *Pfam*. The gene expression raw data obtained using RNA-seq was downloaded from the data base *Sequence Read Archive (SRA)* in *fastq* format. The first stage of our pipeline consisted in gene expression estimation and differentially expressed genes (DEG) selection. In this stage the software tools *Tophat* for read alignment to the corresponding reference genome, *Cufflinks* for transcript assembly and *Cuffdiff* for the estimation of differential gene expression ,were used. The next stage consisted in the construction of the gene co-expression networks where the expression profile for the DEGs was extracted and their correlations were computed using the *R* package *WGNA*. A correlation threshold to determine significant gene co-expression was established employing the *R* package *igraph*. Network visualization was performed with the software tool *Cytoscape*. The third stage, functional patterns discovery in the neighborhood of our genes of interest, was performed using the sequence similarity program *BLAT* to identify homologs and the web tools *agriGO* and *GOrilla* for the GO term enrichment. Finally, Q-PCR experiments were used to validate the analysis.

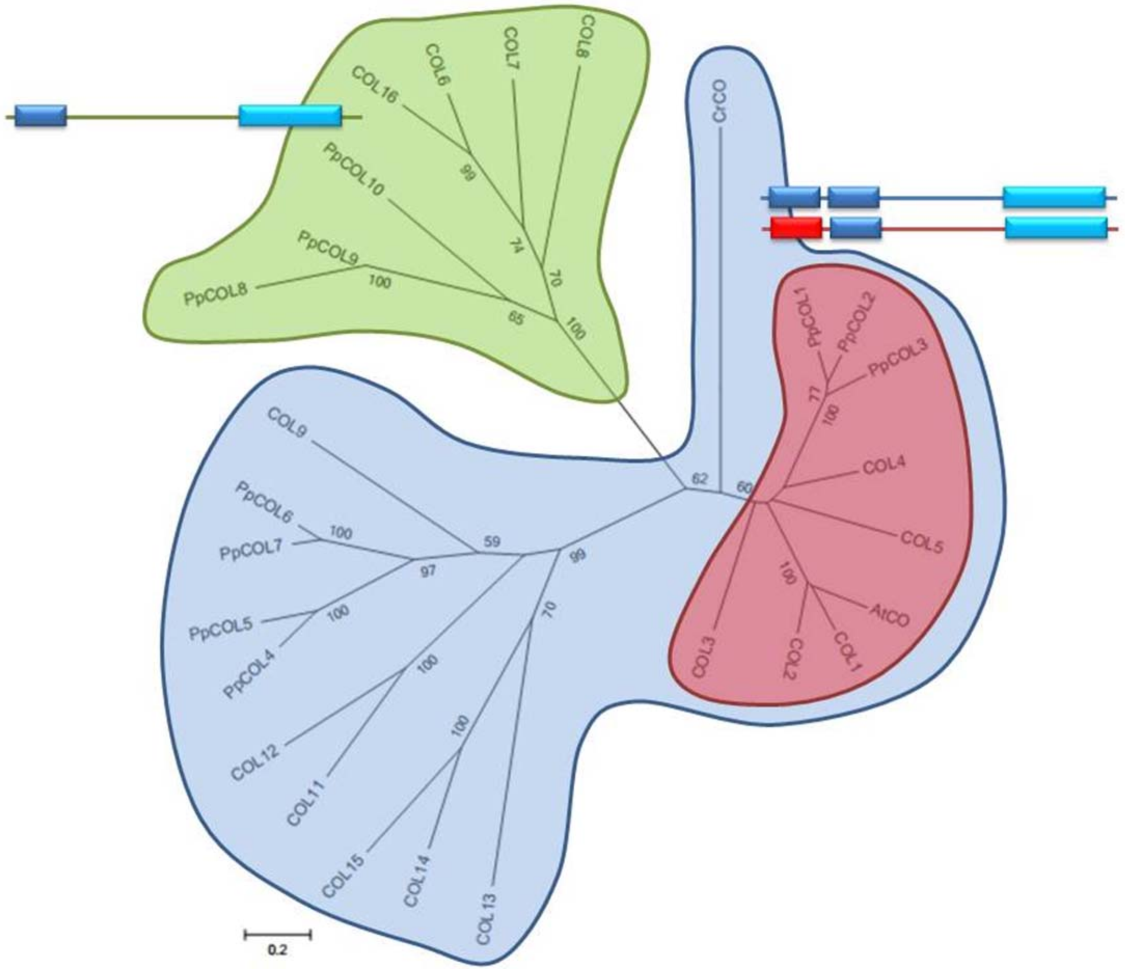

Figure S2. Phylogeny and motif conservation of the COL proteins in *Chlamydomonas*, *Physcomitrella* and *Arabidopsis*. COL proteins can be classified into three different clades descending from CrCO according to this phylogenetic and motif conservation analysis. All COLs present a CCT domain in the C-terminal part but they differ in their N-terminal B-boxes. A first big clade, highlighted in blue, consists of COLs that conserve two similar B-boxes already present in CrCO. This clade includes AtCOLs (COL9, COL13) and PpCOLs (PpCOL4, PpCOL5). COLs in the second clade, highlighted in red (CO, COL1, COL2, PpCOL1) present a first divergent B-box and a second original B-box. Finally, COLs in the third clade highlighted in green, present a single B-box (COL7).

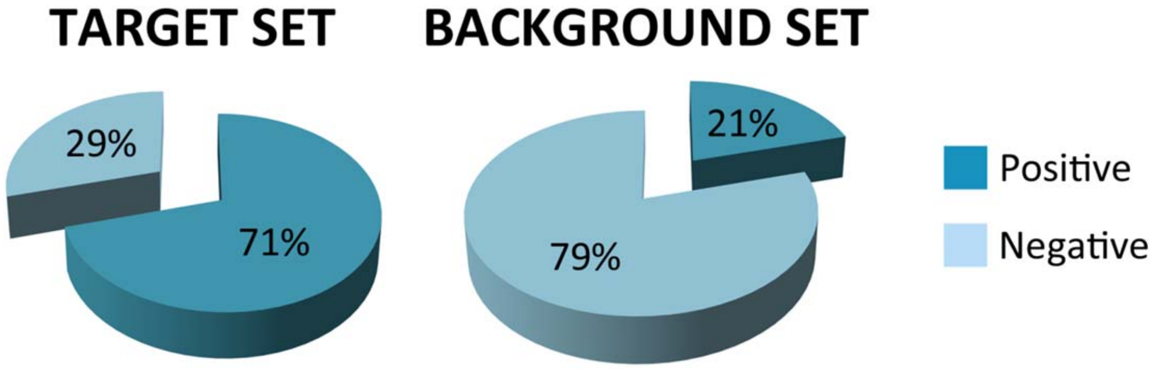

Figure S3. CO binding site enrichment analysis. The gene co-expression network analysis suggests a group of CO target genes (located in its neighborhood) that are often close to CrCO in *Chlamydomonas,* PpCOLs in *Physcomitrella* and other AtCOLs in *Arabidopsis*. In order to corroborate this observation we performed a transcription factor binding site enrichment analysis employing the program *HOMER* for CO the binding sequence CORE (CATN...NCACA). We can observe that these sequences appeared significantly enriched in the target set (left) when compared to a list of background set (right). The enrichment for CORE binding sites in CO co-expressed gene promoters was significant, with a p-value for the Fisher's exact test of 0.0027.
